# Gallic Acid-Chitosan Conjugate Inhibits the Formation of Calcium Oxalate Crystals

**DOI:** 10.3390/molecules24112074

**Published:** 2019-05-31

**Authors:** Moacir Fernandes Queiroz, Karoline Rachel Teodosio Melo, Diego Araujo Sabry, Guilherme Lanzi Sassaki, Hugo Alexandre Oliveira Rocha, Leandro Silva Costa

**Affiliations:** 1 Department of Biochemistry, Universidade Federal do Rio Grande do Norte, Natal, Rio Grande do Norte 59.078-970, Brazil; moacirfqn@gmail.com (M.F.Q.); melo.krt@facenemossoro.com.br (K.R.T.M.); popoh.diego@gmail.com (D.A.S.); 2 Department of Biochemistry and Molecular Biology, Universidade Federal do Paraná, Curitiba, Paraná 81.531-980, Brazil; sassaki@ufpr.br; 3 Instituto Federal de Educação, Ciência, e Tecnologia do Rio Grande do Norte (IFRN), Rio Grande do Norte, Canguaretama, Rio Grande do Norte 59.500-000, Brazil; leandro-silva-costa@hotmail.com

**Keywords:** urolithiasis, kidney stone, polysaccharides, gallic acid grafted chitosan

## Abstract

It has recently been shown that chitosan (Chit) induces the formation of calcium oxalate (CaOx) crystals, which are mainly responsible for the appearance of kidney stones, and this might limit the use of Chit in vivo. Here, Chit was conjugated with gallic acid (Chit-Gal) to decrease the formation of CaOx crystal. This conjugation was confirmed by FTIR and NMR analyses. Chit-Gal contains 10.2 ± 1.5 mg GA per g of Chit. Compared to the control group, Chit increased the number of crystals by six-fold, mainly in the number of monohydrated CaOx crystals, which are the most harmful CaOx crystals. In addition, Chit increased the zeta potential (ζ) of CaOx crystals by three-fold, indicating that Chit was associated with the crystals. These alterations were abolished when Chit-gal was used in these tests. As oxidative stress is related to renal calculus formation, Chit and Chit-Gal were also evaluated as antioxidants using total antioxidant Capacity (TAC), reducing power, ferrous chelation, and copper chelation tests. Chit-gal was more efficient antioxidant agent in TAC (2 times), in ferrous chelation (90 times), and in reducing Power (5 times) than Chit. Overall, Chit-gal has higher antioxidant activity than Chit, does not induce the formation of CaOx crystals. Thus, Chit-Gal has potential to be used as a chit substitute.

## 1. Introduction

Calcium oxalate (CaOx) crystals are involved in the formation of kidney stones, and previous reports show that they account for 70% of the cases of kidney stones [1,2]. Oxalate crystals form naturally during the water reabsorption process in the kidneys as well as during the formation of urine. This reabsorption raises the concentration of the salts, leaving the urine supersaturated with these ions, thereby inducing interaction between the molecules, leading to the formation of crystals [3]. The renal pathways have mechanisms that stabilize the CaOx crystals so they can be excreted easily, thereby preventing their accumulation [4]. However, several factors can lead to a failure of this protection system and induce the formation of oxalate crystals that are difficult to excrete and remain for a longer time in the renal system, allowing them to grow and interact with the renal epithelium [5]. When crystals interact with a renal epithelium cells, they can cause oxidative damage, which can lead to cell death, thereby damaging nearby cells; this initiates a destructive cycle: in the damaged area the crystal adhesion is easier, inducing more oxidative damage, leading to more cell death, and so on [6,7].

Epidemiological data show that 12% of men and 6% of women of the world population will have a case of kidney stones during their lifetime, with a recurrence rate of 70–80% in men and 47–60% in women [8]. In addition, this disease tends to affect an economically active population. It has been estimated that kidney stones result in a loss of more than US$ 2 billion in the USA alone due to general expenses related to the patients, medical care, and hospital costs [9]. In addition to this direct expenditure, there is an indirect loss, with a decrease in the productivity of these individuals, since a third of the patients who were treated for urolithiasis were absent from work. Normalizing by the number of total patients, an average of 19 h of work was lost per person treated [9]. It is important to emphasize that there is no specific drug to prevent or treat kidney stone formation, and the treatments available are not efficient [10,11].

There is a growing search for molecules that can stabilize the forms of oxalate crystals that are more easily eliminated by the kidneys and thus prevent the formation of kidney stones. Furthermore, researchers are also searching for antioxidant compounds that can combat the oxidative stress that occurs during the formation of renal calculi [12]. The molecules recently highlighted in these studies are polysaccharides [5,13,14,15,16].

However, recently we have shown that the polysaccharide chitosan (Chit) may be related to the formation of kidney stones [17]. This paper showed that Chit can induce the formation of CaOx monohydrate (COM) crystals in vitro, which are the major kidney stone-forming crystals [8]. In addition, Zhang et al. [14] and Xia et al. [18] have shown that Chit molecules in the bloodstream undergo renal excretion and have determined that the concentration of Chit in the kidneys increases with time with a maximum concentration 8 h after Chit administration. Hence, the Chit molecule induces the formation of oxalate crystals, and when Chit is administered in vivo, it accumulates in the kidneys.

Chit is an abundant polymer commercially available in different weights and degrees of deacetylation, and it has been extensively studied since the 1970s [19]. For example, in 2018, more than 7000 articles have been published on Chit [20], which was more than the number of articles published for other polysaccharides such as sulfated polysaccharides (659), glycosaminoglycans (578), and gums (2400) [20]. This large number of publications related to Chit is due to the fact that studies on Chit are being conducted in several domains ranging from the purification and characterization of new Chit molecules [21] to the study of their applicability [22] for the production of filter membranes [23], biofilms for food preservation [24], scaffold for bioengineering [25], drug carriers [26,27], and vaccines [28,29], etc. All these potential applications could be impaired if Chit presents a side effect, such as the induction of renal calculi formation.

One method to reduce this adverse effect would be to chemically modify Chit to reduce the formation of CaOx crystals. There are several ways to modify Chit, including depolymerization, sulfation, alkylation, acylation, quarternization, thiolation, and phosphorylation [30]. In addition, these modifications may potentiate the antioxidant activity of chitosan molecules. As for example, chitosan molecules were conjugated with ascorbate and showed high scavenging and chelation abilities [31]. Quaternized Chit derivatives and carboxymethyl also displayed superior antioxidant properties exhibiting dose-dependent reducing power and lipid peroxidation inhibition effect [32]. However, these methods present challenges, including the fragmentation of Chit, the use of expensive and restricted reagents, and requiring multiple reactions, often requiring toxic reagents and competent people to handle them. Furthermore, these methods can generate a large amount of toxic waste for the environment [30].

An alternative to this problem would be the use of green methods to modify Chit, such as the one suggested by Curcio et al. [33]. This method covalently conjugates gallic acid (GA) molecules to Chit molecules, does not produce toxic waste as other methods, like carboxylation [34] and sulfatation [35], and was efficient in modifying some properties of Chit, such as increasing its solubility in water and the antioxidant activity. In addition, it is simple, cheap, and easy method.

Therefore, the main objective of this study was to obtain Chit molecules conjugated with gallic acid (Chit-Gal) using a green method and to evaluate its effect on the formation of CaOx crystals in vitro.

## 2. Results and Discussion

### 2.1. Determination of the Amount of GA Conjugated to Chit

As described in the Methods section, we determined the molecular weight and degree of acetylation of chitosan used herein. The Chit showed 58 kDa and 76 ± 4.8% deacetilation degree (DD).

To first detect the incorporation of GA into Chit, we used the GA dosage test as described in the Methods. An amount of 10.2 ± 1.5 mg GA per g of Chit was found and correspond to grafting yield. When we examine papers that produce gallic acid grafted chitosan using the same method we used, it turns out that molecular weight is a factor that influences the percentage of grafting yield. Cho et al. [36] and Liu et al. [37] used Chit with a molecular mass greater than 200 kDa and they achieved approximately 118 mg/g and 128 mg/g of grafting yield, respectively. However, when we compared our grafting yield with that presented by Curcio et al. [33], we found that although they used a chitosan with a molecular weight (~ 95 kDa) almost twice as large as ours, they obtained 7 mg/g of grafting yield. This indicates that other factors may have influenced our best result. The DD could be this factor. In general, the higher DD the greater the grafting yield. However, Curcio et al. [33] used a Chit molecule with DD of 85%. Therefore, DD would not be the factor that would explain we have achieved a better grafting yield than Curcio et al. [33]. Our suggestion is that during the conjugation process, we used Chit in a concentration five times lower than that used by Curcio et al. [33]. Therefore, our solution of chitosan was less viscous, and consequently, this allowed more efficiently acid gallic conjugation. Liu et al. [37] corroborate with our suggestion. These authors showed that with increasing chitosan concentration there is a decrease in the conjugation of GA with chitosan.

### 2.2. Analysis of the FTIR Spectrum of Chit and Conjugated Chit

**Figure 1:**
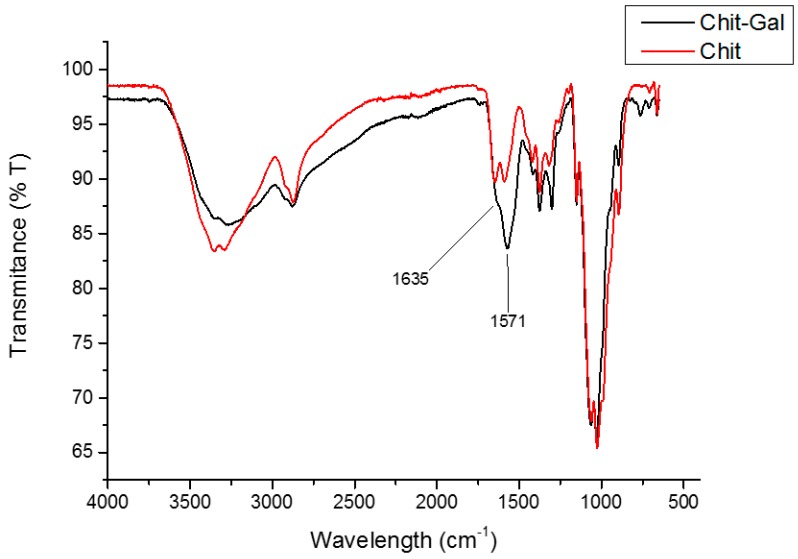
Infrared spectra of unconjugated and conjugated Chit.

The infrared spectrum allows for the quick and efficient identification of signals related to functional clusters present in the molecule. Figure 1 shows the infrared spectra of Chit-Gal (in black) and Chit (in red), with emphasis on the bands at 1635 cm^−1^, 1571 cm^−1^, and 1320–1305 cm^−1^.

The first band was related to the C=O of the amide formed when GA binds to the NH_2_ group of glucosamine [37] and indicates the conjugation of GA with Chit. The second band, which was very intense in the 1571 cm^−1^ region, was obtained because of the overlap of several bands; these were the band relating to vibrations of the aromatic ring of GA, which can arise between 1450 cm^−1^ and 1600 cm^−1^ [37], the band around 1550 cm^−1^ (folding of the N-H of the secondary amide), and the band at 1590 cm^−1^ for the N-H group of the primary amines [38] that were not replaced by GA.

The characteristic bands observed in the Chit and Chit-Gal spectra are summarized in Table 1. In both spectra, there are characteristic bands of polysaccharides [39,40,41], and the specific bands of Chit [38,42,43]. The observed differences between the two spectra were the bands at 1571 and 1635 cm^−1^, which occur only in the spectrum of the conjugated Chit and indicate the presence of GA and its binding to the Chit chain.

### 2.3. NMR Analyses

**Figure 2:**
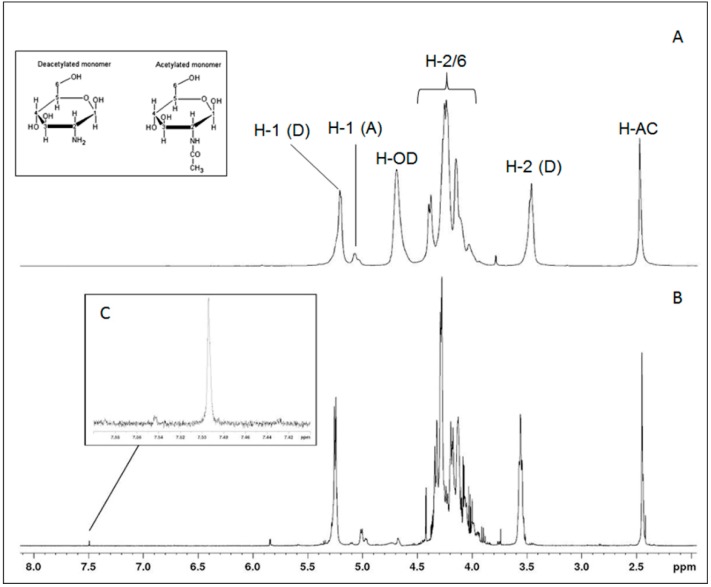
NMR spectra for Chit (**A**) and Chit-Gal (**B**). The identified signals correspond to the typical signs of Chit, present in both samples. H-AC corresponds to the hydrogens of the methyl group of N-acetylglucosamine; D and A correspond to deacetylated and acetylated unities, respectively. H-OD indicates hydrogens of deuterium oxide. Highlighted is the signal at 7.49 ppm (**C**), present only in the Chit-Gal spectrum and corresponds to the hydrogen from the aromatic ring.

The ^1^H-NMR spectra of Chit and Chit-Gal are shown in Figure 2. In the region from 3.90 to 4.45 ppm, the signals referring to the hydrogens 2, 3, 4, 5, and 6 of the aldohexoses overlap, and therefore it was impossible to identify them separately. The signals of the anomeric hydrogen (H1) can be clearly identified at 5.25 and 5.01 ppm for glucosamine (D) and *N*-acetylglucosamine (A), respectively. The signal at 2.45 ppm indicated the hydrogens of the methyl group of N-acetyl-glucosamine (H-AC), and the signal at 3.56 ppm marked the glucosamine H2 (D). All of these peaks were characteristic of Chit and have also been previously reported [44,45,46].

A signal at 7.49 ppm can be observed in Figure 2, which was not present in the spectrum of unconjugated Chit. The region between 7.00 to 9.00 ppm was characteristic of aromatic compounds, which indicates, in this case, the presence of an aromatic group bound to Chit. This same signal was also observed by Liu et al. [37] and Cho et al. [36] when they analyzed Chit-Gal. Thus, this signal at 7.49 ppm confirms the binding of GA to Chit, thereby corroborating the FTIR spectra.

### 2.4. Apparent Molecular Weight Determination

The conjugation process used here has as its first step the creation of regions in the Chit molecule that can bind covalently with GA. Therefore, initially Chit reacted with H_2_O_2_ and ascorbic acid, and later with GA (see the Methods section). This contact with H_2_O_2_ can induce the Chit molecule breakdown (depolymerization) [47]. Therefore, the apparent molecular weight of Chit-Gal was determined by high performance size exclusion chromatography (HPSEC) and the value obtained was 53 kDa. This data shows that during the conjugation process the Chit molecule had its mass decreased by 10% (~5 kDa). In addition, we also determined the apparent molecular weight of Chit (Chit-P-AA) when it was only exposed to H_2_O_2_ and ascorbic acid for 24 h without the presence of GA. Chit-P-AA showed an apparent molecular weight of 52.5 kDa. This difference between the molecular weight of Chit-Gal and Chit-P.AA is due to the GA molecules conjugated to Chit-Gal.

This depolymerization of chitosan after the GA conjugation process was also previously reported when a 98 kDa chitosan molecule was conjugated to GA for biofilm formation. In this case, the depolymerization of chitosan was around 25% [48]. The rate of depolymerization of chitosan during AG conjugation is influenced mainly by two factors: size of the chitosan molecule, the larger the molecule the greater the rate of fragmentation, and the amount of peroxide used in the conjugation process, the greater the amount of peroxide higher the rate of depolymerization [47] This may explain the lower level (~10%) of depolymerization we obtained with Chit-Gal.

### 2.5. Formation of Crystals

#### 2.5.1. Analysis of the Formation Profile for CaOx Crystal Formation In Vitro

In vitro crystal formation tests were performed to evaluate the sample ability to inhibit or stimulate the formation of these crystals. The natural formation of the crystals takes place in three different stages: nucleation, growth, and aggregation. The first stage consists of electrostatic attraction: the ions present in solution attract each other and form the first nanocrystals, these nanocrystals are the nuclei that attract more ions and start the second phase, the growth phase, in which the the nanocrystals increase in size. Within the solution, the crystals formed begin to collide and aggregate, forming larger crystals until they are large enough to precipitate, this is the third and final phase, the aggregation phase [49].

An analysis of the crystal in vitro formation profile identifies these phases by separating them at two distinct moments: the ascending phase and the descending phase. In the ascending phase there is a continuous increase in absorbance, this moment corresponds to the nucleation and growth phases of the natural formation of the crystals. The decrease in absorbance values marks the beginning of the second moment. This moment is related to the aggregation phase and following crystals precipitation, this phase ends when absorbance stability is achieved.

**Figure 3:**
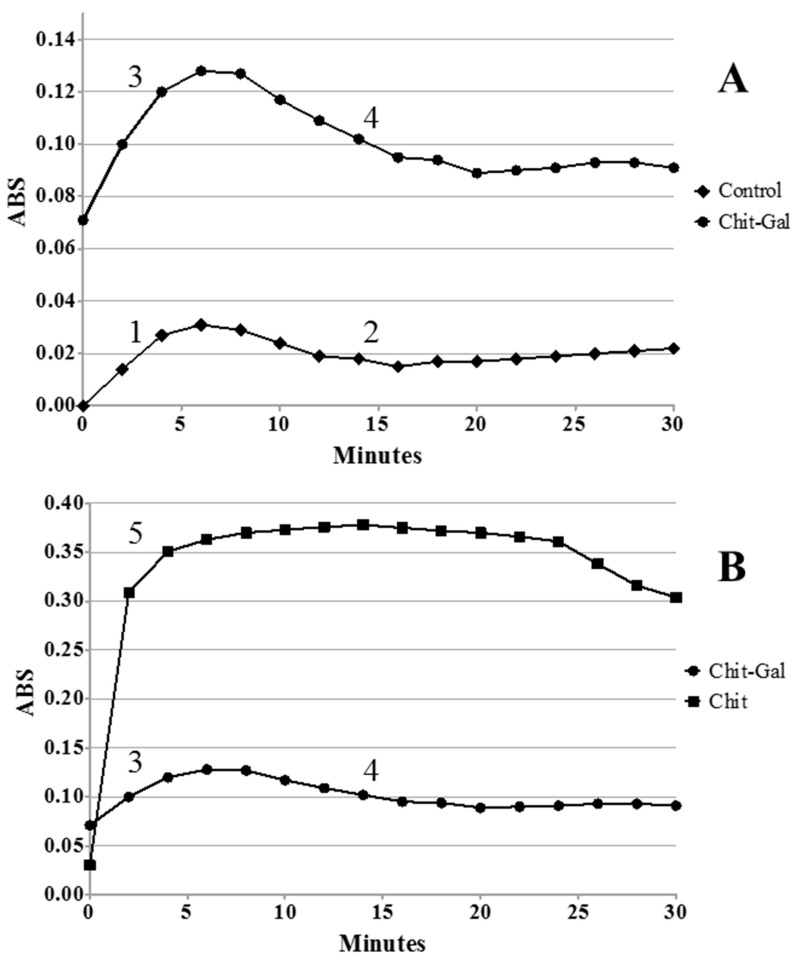
Formation profile of CaOx crystals. **A**. Formation profile in control condition and in the presence of chit-gal. **B**. Formation profile in the presence of chit-gal and Chit. 1, 3, and 5 indicate nucleation/growth phases. 2 and 4 indicate aggregation phase.

Therefore, we evaluated whether the samples influenced the formation of crystals as well as the phase that is influenced. Figure 3 summarizes the results concerning the formation of CaOx crystals in the presence or absence of Chit or Chit-Gal. In the control group as well as in the presence of Chit-Gal, the two distinct phases of crystal formation were observed: the nucleation/growth (Figure 3A, numbers 1 and 3) and aggregation (Figure 3A, numbers 2 and 4), whereas in the presence of Chit there is only the nucleation/growth phase (Figure 3B, number 5), since the absorbance, after 30 min, does not stabilize. In addition, in the presence of Chit-Gal, a 4-fold increase of the absorbance values was observed when compared to the control, whereas in the presence of Chit, a 10-fold increase in absorbance values were obtained. These results were consistent with those obtained by Queiroz et al. [17], who used the same unconjugated Chit used in this study. These authors demonstrated that when the crystals were formed in the presence of Chit, a 15-fold increase in absorbance was observed. In addition, the authors did not observe the aggregation phase until the end of the experiment. This implies, that the addition of only 1% of GA in the Chit structure was enough to decrease its stimulating towards the formation of GA crystals.

#### 2.5.2. Morphology of Crystals Produced In Vitro

**Figure 4:**
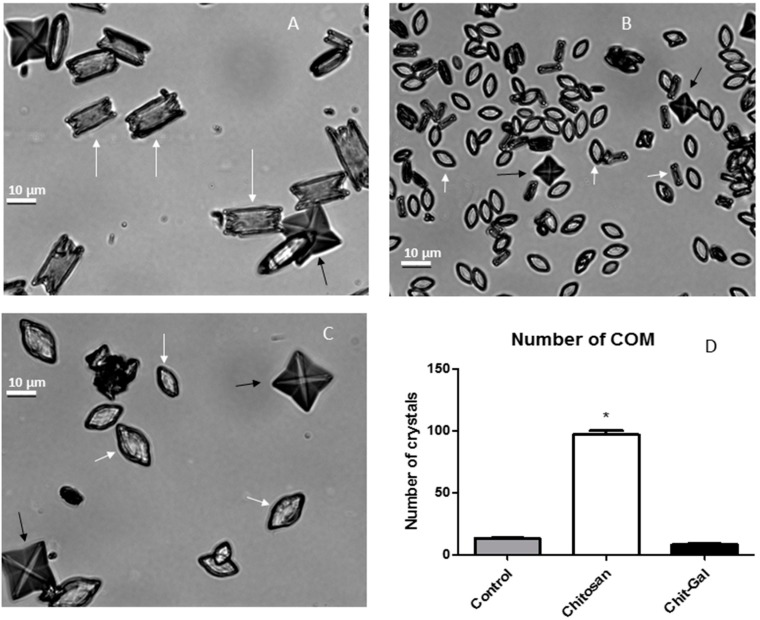
Morphology of crystals formed in the presence and absence of chitosan (Chit). (**A**) Absence of Chit (Control), (**B**) Chit, and (**C**) Chit-Gal. (**D**) Number of monohydrate crystals (COM) formed by field. White arrows, COM; black Arrows, dihydrate crystals (COD). Scale bar: 10 μm. Different symbols indicate significant difference (*p* < 0.05).

Three types of CaOx crystals can be formed, and the presence of these different crystals in the urine of patients results in different prognoses. The most commonly found crystal in the urine of healthy and non-calculating patients are dihydrate crystals (COD), which has a pyramidal or pinwheel morphology, is more easily excreted, but needs to be stabilized by physiological mechanisms [5]. When it is not stabilized, it converts to monohydrate crystals (COMs) [50]. COMs are more stable crystals and are found in the urine of renal calculi patients, have a rectangular morphology, and are strongly associated with urolithiasis [50]. When trihydrated CaOx (COT) crystals are formed, they have a druse-like morphology, they are rarely found in the urine of patients because these crystals are very unstable and rapidly converts to other types of crystals [51]. Evaluation of crystal morphology in vitro allows us to understand what type of crystal is formed and what influence the sample has on the size and morphology of these crystals. The CaOx crystals formed in the presence or absence of different polysaccharides can be observed in Figure 4. COM and COD crystals were present in all conditions tested with the different Chit samples. It was evident that Chit induced the formation of a greater number of crystals, where 94.5 ± 14.35 crystals per field were observed, which was 6 times greater than the 15 ± 3.33 crystals per field observed in the control group. However, 9.25 ± 4.04 crystals per field were observed in the presence of Chit-Gal, like that observed in the control group.

Next, the type of crystal that was formed was evaluated. The presence of COT crystals (COT), was not observed under the conditions tested. However, the number of COM formed is shown in Figure 4D, where 12.63 ± 2.65 COM per field were observed in control group, which represents approximately 84% of the crystal population observed. In the presence of Chit, 92.1 ± 12.92 COM per field were observed, representing a 7-fold increase. It is worth noting that these crystals represent approximately 97% of the total crystals observed. It has been previously reported that COM induce a greater amount of damage and cell death compared to COD [2]. The binding of GA to Chit reversed this undesired effect of Chit: in Chit-Gal group 6.91 ± 2.60 COM per field were observed, corresponding to 75% of the total number of crystals and similar to the values observed in the control group. In summary, the presence of Chit-Gal reversed the Chit effect both with respect to the number of crystals and with respect to the type of crystal formed.

Additionally, the COM-type produced in the presence of Chit and Chit-Gal (Figure 4B,C) were somewhat different from those observed in the control: these crystals had an elliptical morphology. Another observation made was that in the presence of Chit, the crystals formed were smaller. To confirm this, the crystals were measured, revealing that the average sizes of the crystals formed in the presence of Chit (COM, 7.96 ± 1.35; COD, 10.88 ± 5.08) were lower than that observed for the control (COM, 12.63 ± 2.65; COD, 21.32 ± 3.08), while the size of the crystals formed in the presence of Chit-Gal was comparable to that observed for the control (COM, 16.39 ± 6.12; COD, 20.60 ± 9.84).

The formation of the CaOx crystals is spontaneous and depends on the aggregation of the crystal forming ions and the nuclei formed from this aggregation [45,50]. It is noteworthy that these two processes are always dependent on the charges of the ions in solution, which leads to the formation of organized crystals with more positively charged faces and others more negatively charged [47,48,52,53]. Several negatively charged macromolecules can interfere in this process by modifying the morphology and/or size of the CaOx crystals [49,54]. However, there is still no consensus regarding the mechanism of action of these molecules [49,55]. In relation to positively charged molecules, such as chitosan, studies are insipid, but nevertheless Gul and Rez [50,55] state that this type of molecule is expected to be electrostatically attracted to the more negative portion of the crystals, and thus interfere in the formation of these crystals. The amine groups present in Chit give it a positive charge at the pH of the test (close to the pH of the urine), so we suggest that Chit interacts with the more negative portions of the crystal during its formation, thus altering the electrostatic charge equilibrium of the crystal and consequently altering its growth. Consequently, these crystals with altered electrostatic equilibrium have difficulty to aggregate, creating a tendency to form smaller crystals with unconventional morphology. In addition, as the crystals do not aggregate, many small crystals are formed (Figure 4B). This corroborates with data previously shown by Queiroz et al. [17] and with the proposed mechanism of action for several negative molecules [15,56].

When Chit-Gal was used, larger crystals were formed (Figure 4C). We believe that this happened since one of the main binding points of AG in Chit-Gal is exactly the amine groupings [33,36,37]. This binding decreases the overall charge of the chitosan molecule, causing its interaction with the crystals to be smaller, and consequently to have a crystallization (Figure 4C) more similar to that observed in the control group (Figure 4A).

#### 2.5.3. Evaluation of Zeta Potential

**Figure 5:**
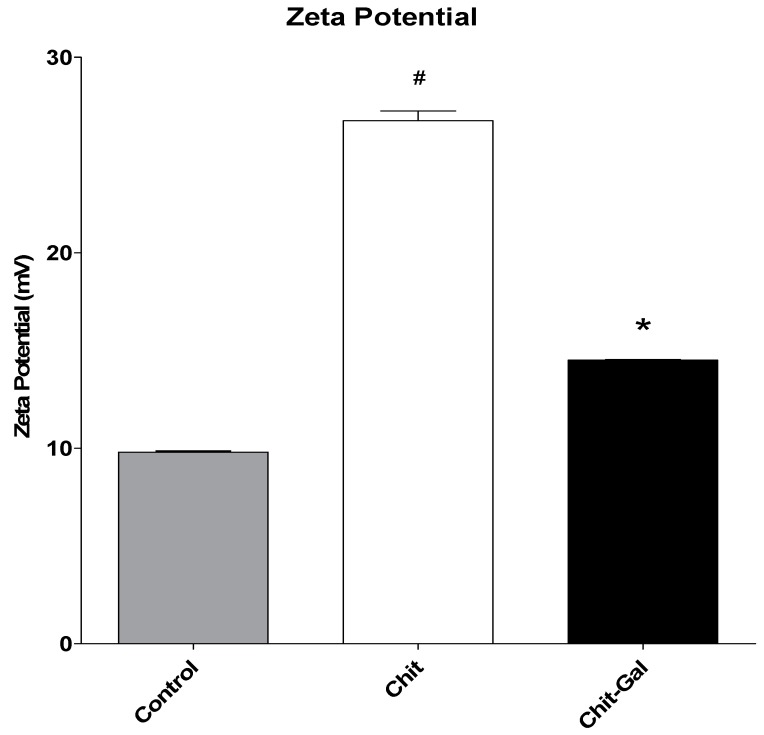
Zeta potential of the crystals formed in the presence of different chitosans. Different symbols indicate a significant difference (*p* < 0.05).

The zeta potential test measures the surface charge of the crystals, allowing the evaluation of how the sample loads influence the total charge of the crystal. The value of the zeta potential of the crystals formed in the presence of the polysaccharides is shown in Figure 5. It can be noted that in the presence of Chit, the zeta potential of the samples increased. This increase, may be related to the fact that Chit is a cationic polymer, mainly due to the positive charge of its amine groups [57]. Despite this, the increase in the zeta potential observed in the presence of Chit-Gal (14.5 ± 0.1 mV) was lower than that observed with Chit (26.7 ± 0.98 mV), and the zeta potential of the former was close to that of the control condition (9.8 ± 0.14 mV).

We hypothesized that Chit-Gal would induce a different conformation than Chit, as it contained GA, and therefore, when Chit-Gal interacts with the crystals formed, it would exhibit fewer positively charged groups than Chit. In addition, the FTIR data suggests that the main binding site of GA were the NH_2_ groups of Chit. Therefore, Chit-Gal has fewer NH_2_ groups, and therefore less positive charge than Chit. Consistent with Cho et al. [36], one of the main regions of entry of GA into Chit is the NH_2_ group on carbon 2, and therefore the zeta potential of the crystals in the presence of Chit-Gal was smaller than that in the presence of Chit. We believe that a reduction in the surface charge of the crystals was responsible for the formation of larger and fewer crystals when Chit-Gal was used. This mechanism of action was likely observed by Melo et al. [15], who induced the formation of crystals in the presence of sulfated polysaccharides, and noted a reduction in the surface charge of the crystals and consequently lower crystal formation.

### 2.6. Evaluation of Antioxidant Activities of Chit

#### 2.6.1. Total Antioxidant Capacity (TAC)

**Figure 6:**
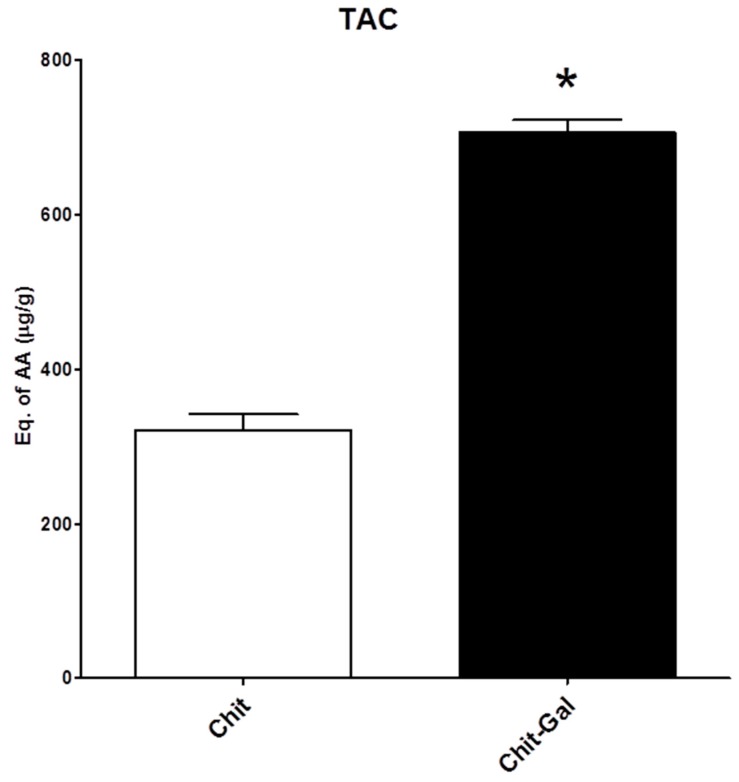
Total antioxidant capacity of the samples, in μg of ascorbic acid/g sample. *indicates *p* < 0.05.

The TAC test measures the ability of a molecule to donate electrons in acid media. Figure 6 shows the total antioxidant capacity of the Chit samples. Chit presented activity at approximately 300 μg ascorbic acid (AA)/g of sample, while Chit-Gal showed higher activity than 600 μg AA/g of sample, indicating that the conjugation of GA to Chit was able to double the TAC of the polysaccharide. These values were low when compared to other molecules, such as sulphated [58] and neutral polysaccharides [39], where the minimum observed activity was about 10 mg AA/g of sample. However, these results were an improvement over those published by Curcio et al. [33], who conjugated GA molecules to Chit, but did not observe an increase in the TAC of the molecule.

#### 2.6.2. Iron Chelation

**Figure 7:**
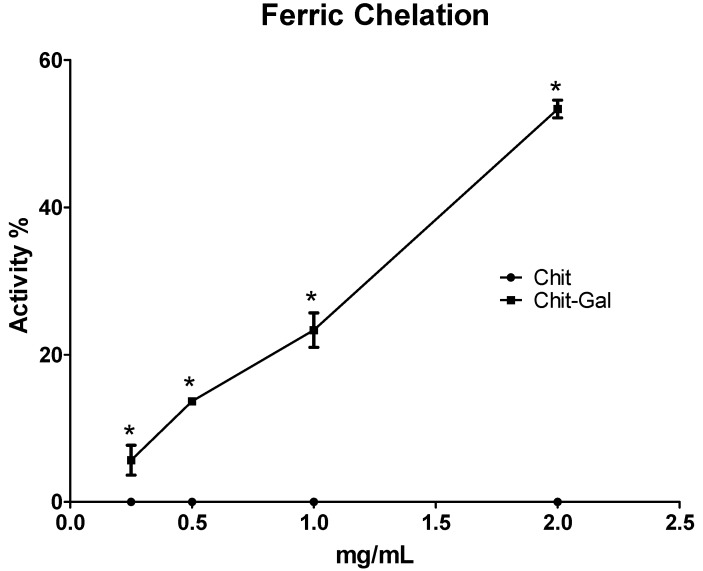
Iron chelating activity. The * indicates statistical difference (*p* < 0.05). EDTA was used as a positive control and had 100% activity at a concentration of 0.025 mg/mL.

Iron is related to propagation reactions in the oxidative stress damage, and the ability to chelate this metal can prevent propagation and reduce the damage caused by reactive species. The data obtained in the iron chelation test are shown in Figure 7; this data shows that Chit does not have the ability to chelate iron. There have been many reports on the iron chelating activity of Chit, but the activities were not as prominent, never exceeding 35% [59,60,61]. An important factor related to the iron chelating activity of Chit is a high degree of deacetylation (more than 90%) associated with the small size of the molecule. Although the Chit used in this study was 58 kDa, the association of this size with the 76% deacetylation value was insufficient for this Chit sample to present iron chelating activity, which corroborates with that shown with other Chit samples with these similar characteristics [60,61].

However, Chit-Gal can chelate iron. At the maximum concentration tested (2 mg/mL), Chit-Gal showed a 50% chelation activity of this ion. Interestingly, previous studies have shown that GA was not a good iron chelator [62,63], and the Chit used in this study also did not present any activity; however, the conjugation of GA to Chit conferred activity that was superior to the individual activity of the two molecules. A similar result was observed by Xie et al. [64], who used Chit conjugated to GA molecules that resulted in a molecule with 4-fold more iron chelating capacity than the original Chit.

In humans, iron have important physiological functions, even when it is complexed to proteins such as heme enzymes, myoglobin in muscles, and hemoglobin [65]. However, humans do not have any inherent mechanisms to excrete metal, and this can lead to metal accumulation and, which ultimately leads to diseases. There have been several reports that demonstrated the relationship between iron accumulation in diabetes [66], liver cancer, and osteoporosis among other disorders [67]. To date, there has been no cure for patients with iron overloads. Thus, iron levels are controlled by phlebotomy or administration of iron chelators [66]. Thus, the fact that Chit-Gal has iron chelating activity suggests that it is a possible treatment for patients with iron accumulation-related diseases.

#### 2.6.3. Copper Chelation

**Figure 8:**
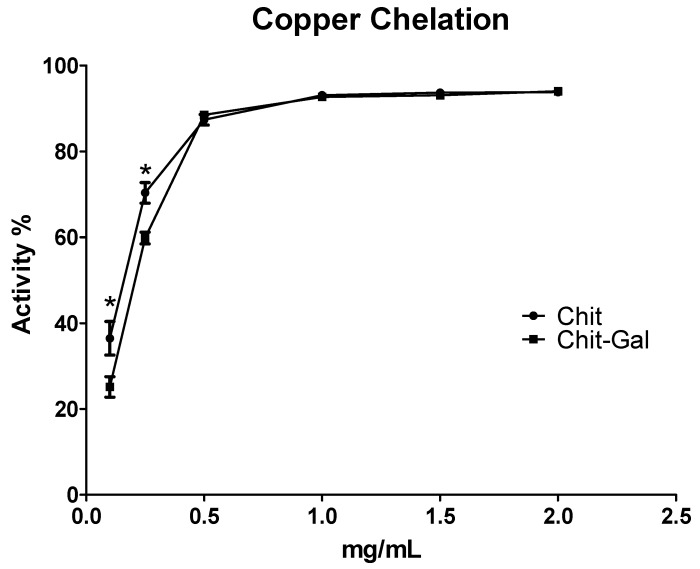
Activity of copper chelation of the samples. The * indicates statistically different values (*p* < 0.05). EDTA was used as a positive control and had 100% activity at the concentration of 0.05 mg/mL.

Like iron, copper is also involved in the spread of damage caused by free radicals. Shown in Figure 8 is a comparison of the chelating activities of Chit and Chit-Gal. At low concentrations (up to 0.25 mg/mL), Chit exhibited significantly greater activity than Chit-Gal. At concentrations above this threshold, no significant differences were identified between Chit and Chit-Gal. The activity for both samples was notable and increased in a dose-dependent manner; it increased from 60% chelation at a concentration of 0.25 mg/mL to close to 100% chelation at 1 mg/mL. The relationship between Chit and copper has been widely reported, and the interaction capacity between these molecules has been applied in different ways [68,69]. However, the activity was different between all Chit molecules. For example, Monteiro and Airoldi [70] were unable to obtain copper chelation activities greater than 60%, even at higher concentrations (4 mg/mL) than those used in our study. This shows that the conjugation with GA may be useful to potentiate the chelating action of Chit molecules.

At low concentrations, the lower copper chelating ability observed with Chit-Gal can be justified by a reduction in free amine groups. This grouping was mainly responsible for the interaction of Chit with copper [68], and consequently for its chelating action on this metal. In the conjugation process, one of the GA entry points in Chit was the free amine group, such that the conjugated Chit had fewer free amines, leading to a lower copper chelating capacity at lower concentrations [37].

#### 2.6.4. Reducing Power

**Figure 9:**
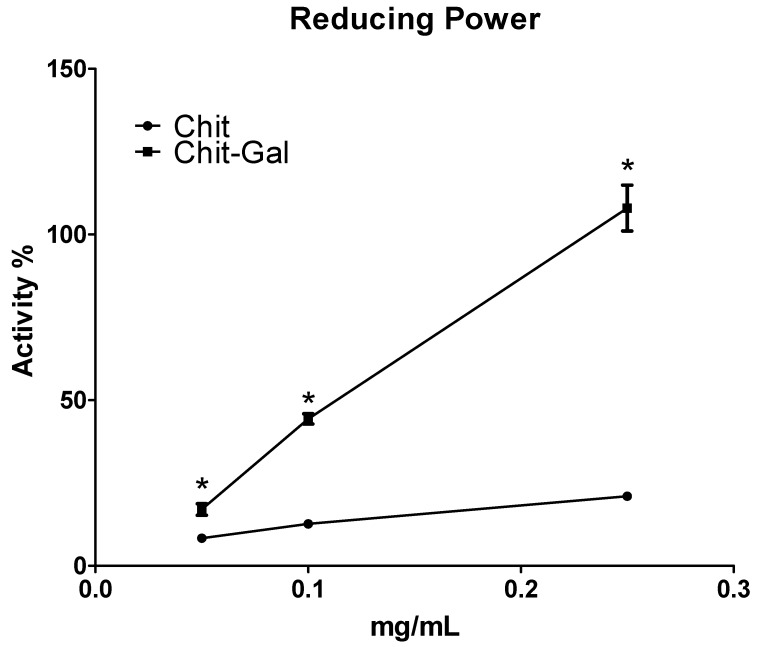
Reducing power of the samples. The * indicates a statistical difference (*p* < 0.05).

The reducing power test evaluates a molecule’s ability to donate electrons. Figure 9 shows the reducing power obtained for the samples. Chit presented with 20% reducing power at the maximum concentration evaluated, and GA conjugated to Chit conferred 100% activity. The activity observed for Chit was similar to that observed with other Chit molecules [42,59,71]. Conjugation of GA resulted in a significant increase in observed activity. GA shows significant activity in this test [72], and at 250 μg/mL Chit-Gal, approximately 2.5 μg/mL of GA was present. Although the mass of this molecule would suggest significant activity, this alone does not justify the 100% activity observed in the test. Thus, the activity observed in this test can be justified by the connection between the two molecules, which led to the formation of a molecule with a reducing power that combined the potentials of both molecules. Xie et al. [64] also reported that conjugation of GA to Chit resulted in a greater reducing power than the individual precursors, which corroborates data obtained in our study.

## 3. Materials and Methods

### 3.1. Materials

Chitosan, gallic acid, L-ascorbic acid, Folin-Ciocalteu reagent and dextran standards was purchased from Sigma-Aldrich (St. Louis, MO, USA). Other solvents and chemicals used in this study were of analytical grade.

### 3.2. Conjugation of Gallic Acid onto Chitosan

The conjugation used a redox method as first described by Curcio et. al. [33] with brief modifications. First 500 mg of chitosan was dissolved to 10 mg/mL in acetic acid water solution (2% *v*/*v*). Then 1 mL of 1 M H_2_O_2_ and with 0.054 g of ascorbic acid were added to the solution. After 30 min, 1.4 mmol GA was introduced to the reaction and incubated for 24 h, at room temperature. The polymer was then dialyzed with a 12 kDa membrane cut off and freeze-dried. The sample was named Chit-Gal.

### 3.3. Quantification of Gallic Acid

The amount of gallic acid conjugated to chitosan was determinate using the Folin-Ciocalteau as described by Cho et al. [36]. Briefly, 40 µL of each sample (1 mg/mL) was mixed with 200 µL of FolinCiocalteau reagent and 1160 µL of distilled water for 3 min, followed by 600 µL 20% sodium carbonate (Na_2_CO_3_). The mixture was shaken for 2 h at room temperature and then a 200 µL of the mixture was added to each well of a 96-well microplate. Absorbance was measured at 720 nm using a microplate reader (SpectraMax^®^ M2/M2e, Molecular Devices, San Jose, CA, USA). Gallic acid was used as a standard.

### 3.4. Fourier Transformed Infrared Spectroscopy (FTIR)

A Nexus 470 ESP FTIR spectrometer (Thermo Nicolet, Madison, WI, USA) was used to obtain the infrared spectra between 500 and 4000 cm^−1^, of a tablet containing mixed KBr and different samples (5 mg). Thirty-two scans at a resolution of 4 cm^−1^ were evaluated and referenced against air.

### 3.5. Nuclear Magnetic Resonance (NMR) Spectroscopy

The samples (50 mg) were dissolved in 800 μL of acidified deuterium oxide (D_2_O). NMR spectra (1H) were obtained in a Bruker Avance III HD 600 MHz spectrometer (Bruker BioSpin Corporation, Billerica, MA, USA) equipped with a 5-mm inverse quadruple resonance probe (QXI) at 70 °C. The chemical shifts were expressed in δ relative to sodium trimethylsilyl propionate (TMSP) at δ = 0.00 in accordance with IUPAC recommendations. For determining the DD sample, we used the integrals of the peaks observed in the anomeric region of ^1^H-NMR as demonstrated by Lavertu et al. [46], which applied to our chitosan. The formula used was: DD (%) = (H1D/(H1D + H1A)) × 100.

### 3.6. Determination of Chitosan Molecular Weight

The molecular weight of chitosan was determined by high performance size exclusion chromatography (HPSEC) (GE Healthcare Bio-Sciences, Pittsburgh, PA, USA) on TSK-Gel^®^ 3000 (30 cm × 0.75 cm), with a column temperature of 60 °C. The chitosan was eluted with 0.2 M NaCl in 0.05 M acetate buffer, pH 5.0, at a flow rate of 1.0 mL/min and detected by a refractive index detector. The column was calibrated using different dextrans (10; 47; 74; 147 kDa) purchased from Sigma (St. Louis, MO, USA).

### 3.7. Calcium Oxalate Crystallization Assay

The effect of polysaccharide in the crystallization of calcium oxalate was spectrophotometrically measured for 30 min at 620 nm, as described by Zhang et al. [56].This assay is based on quantification by the optical density of metastable solutions of Ca^2+^ and oxalate, by means of a mixture of calcium chloride (8 mM) and sodium oxalate (1 mM), 200 mmol/L of sodium chloride and 10 mmol/L of sodium acetate. The concentrations of compounds present in this mixture are close to the physiological urinary concentrations. The CaCl2 (1.0 mM) solution was constantly stirred at 37 °C, either in the absence or the presence of different concentrations of chitosan (100, 50 and 25 μg/mL). After obtaining a stable baseline, crystallization was induced by the addition of a solution of Na2C2O4 (1.0 mM) to achieve final concentrations of 4 Mm of calcium and 0.5 mmol/L of oxalate. The data is presented as absorbance versus time (min).

### 3.8. Crystal Morphology Analysis

Crystal formation was induced in the presence or the absence of chitosan or dextran (100 μg/mL). After 30 min, the solutions were centrifuged (5000× *g*), and the supernatant was discarded. The crystals were then suspended in 0.5 mL of distilled water and a part of 0.1 mL was put on a histological blade and taken to a microscope. The crystal morphology was analyzed in 10 randomly selected fields at 60× magnification. Images were captured from different fields. Three different experiments were performed.

### 3.9. Zeta Potential (ζ) Measurements

The crystals were induced to form in the presence or absence of chitosan or sodium citrate (0.25 mM). After 30 min, the solutions were centrifuged (5000× *g*). The crystal concentrate was then suspended in 1.5 mL of water, and the zeta potential of the ζ samples was obtained using a Zeta Plus^®^ analyzer (Brookhaven Instruments, Holtsville, NY, USA).

### 3.10. Antioxidant Activity

#### 3.10.1. Determination of Total Antioxidant Capacity

This assay was carried out as described by Costa et al. [58]. It is based on the reduction of Mo (VI) Mo (V) by sample and subsequent formation of a phosphate green complex/Mo (V) at acid pH. Tubes containing chitosan and reagent solution (0.6 M sulfuric acid, 28 mM sodium phosphate and 4 mM ammonium molybdate) were incubated at 95 °C for 90 min. After the mixture had cooled to room temperature, the absorbance of each solution was measured at 695 nm against a blank. Total antioxidant capacity was expressed as ascorbic acid equivalent.

#### 3.10.2. Ferrous Chelating

The method uses the ferrozine and FeCl2 complex to determine the antioxidant capacity, as described according to Melo-Silveira et al. [73]. Briefly, the reaction mixture contained the different samples (from 0.1 to 2.0 mg/mL), FeCl2 (2 mM) and ferrozine (5 mM). The mixture was homogenized. After 10-min incubating at 37 °C the absorbance was read (562 nm) in a microplate reader.

#### 3.10.3. Copper Chelating

The ability to chelate the copper ion from the extracts was determined by the method described by Melo et al. [74]. Pyrocatechol violet, the reagent used in this assay, can associate with certain cations, such as aluminum, copper, bismuth and thorium. In the presence of chelating agents, this combination is not formed, resulting in decreased staining. The test is performed in 96-well microplates with a reaction mixture containing different concentrations of samples (0.1–2 mg/mL), pyrocatechol violet (4 mM) and copper II sulfate pentahydrate (50 mg/mL). All wells were homogenized with the aid of a micropipette, and the solution absorbance was measured at 632 nm.

#### 3.10.4. Reducing Power

The reducing power was quantified according to the methodology described by Presa et al. [75]. The test samples (1 mL) in different concentrations (0.05–0.25 mg/mL) were mixed in a phosphate buffer (0.2 M, pH 6.6) with potassium ferricyanide (1%) and incubated for 20 min at 50 °C. The reaction was interrupted by the addition of TCA (trichloroacetic acid) to 10%. Subsequently, distilled water and ferric chloride (0.1%) were added to the samples. Readings were taken at 700 nm. The data were expressed as a percentage of the activity shown by 0.1 mg/mL of vitamin C, which corresponds to 100%.

### 3.11. Statistical Analysis

All the data are expressed as the mean ± standard deviation (n = 3). To test the difference between results, the ANOVA test was performed. The Student–Newman–Keuls test (*p* < 0.05) was used to solve similarities found by the ANOVA. All tests were performed in GraphPad Prism 5 (GraphPad Software Inc., La Jolla, CA, USA).

## 4. Conclusions

In the present study, we evaluated whether the conjugation of Chit with GA using a green method would yield a molecule that would interfere less in the formation of CaOx with better antioxidant activity. Conjugation was confirmed by FTIR and NMR spectroscopy analyses, and indicative signals of GA binding to Chit molecules were observed. It was also observed that Chit increased the formation of CaOx crystals in vitro, and that Chit-Gal was able to reduce the amount of crystals, like the levels observed in the control. The antioxidant potential of Chit was improved after conjugation in all tests except the copper chelation test. In summary, the results show that Chit-Gal is a promising antioxidant agent without showing the effect on CaOx crystal formation. Therefore, Chit-Gal has the potential to be used as a Chit substitute in several applications. In addition, further studies to prove chit-gal has the same physico-chemical properties as chitosan are needed.

## Figures and Tables

**Table 1:** Main bands observed on the FTIR spectra of unconjugated and conjugated chitosan.

Signal (cm^−1^)	
660	Presence of NH_2_ Groups
1028 and 1066	Streching of C-O bond
1153	Asymmetric stretching of the C-O-C bridge
1375	CH_3_ symmetrical deformations
1423	CH_2_ bending
1589	N-H bending of the primary amine
1645	C=O stretching of amide I
2877	C-H asymmetric stretching
2921	C-H symmetric stretching
3291 to 3361	N-H and O-H stretching and the intramolecular hydrogen bonds
1571	Vibrations of the aromatic ring from GA
1635	C=O stretching of amide II
